# The Association and Significance of MDM2 and NF-κB Protein Expression in Multiple Myeloma

**DOI:** 10.3390/medicina61111948

**Published:** 2025-10-30

**Authors:** Marija Stanić Damić, Aron Grubešić, Zinaida Perić, Nives Jonjić, Irena Seili-Bekafigo, Goran Hauser, Nina Jajaš, Toni Valković

**Affiliations:** 1 Department of Internal Medicine, Division of Hematology, Clinical Hospital Center Rijeka, Krešimirova 42, 51000 Rijeka, Croatia; aron.grubesic@uniri.hr (A.G.); zinaida.peric@uniri.hr (Z.P.); goran.hauser@uniri.hr (G.H.); nina.jajas@fzsri.uniri.hr (N.J.); 2 Faculty of Medicine, University of Rijeka, 51000 Rijeka, Croatia; nives.jonjic@uniri.hr (N.J.); iseilibe1@gmail.com (I.S.-B.); 3 Department of Pathology and Cytology, Clinical Hospital Center Rijeka, 51000 Rijeka, Croatia; 4 Faculty of Health Studies, University of Rijeka, 51000 Rijeka, Croatia; toni.valkovic@uniri.hr; 5 Specialty Hospital Medico, 51000 Rijeka, Croatia

**Keywords:** bortezomib, biomarker, multiple myeloma, MDM2, NF-κB, protein expression, renal impairment

## Abstract

*Background and Objectives*: This pilot study examines the expression of MDM2 and NF-κB proteins in CD138-positive plasma cells in patients with multiple myeloma treated with bortezomib-based therapy, exploring their possible interplay and correlations with clinical and selected prognostic factors. *Materials and Methods*: We analyzed MDM2 and NF-κB protein expression via double immunohistochemical staining of bone marrow trephine biopsies obtained at diagnosis and after bortezomib-based therapy. Statistical analyses were performed to assess the significance of changes in protein expression before and after therapy and their association with clinical and prognostic parameters. *Results*: Key findings revealed a positive correlation between MDM2 and NF-κB, as well as a significant decrease in their expression following therapy. Decreased NF-κB expression seems to be an independent prognostic factor for improved renal function. *Conclusions*: The results demonstrated for the first time the in vivo protein expression of MDM2 in the bone marrow of patients with multiple myeloma, as well as the possible effect of bortezomib on the expression of this protein in the microenvironment of multiple myeloma.

## 1. Introduction

Multiple myeloma (MM) is a hematological malignancy characterized by the expansion of neoplastic plasma cells in the bone marrow and is associated with high mortality. Despite evolving myeloma-targeted therapeutic regimens and significant improvements in patient survival these therapies are still not curative. Myeloma cells survive and disturb their surrounding bone marrow microenvironment to induce therapeutic resistance and hamper immune response. Altered expressions of tumor suppressors, such as p53, have been described in a significant number of patients, which contributes significantly to disease progression [1]. However, the key regulatory elements that control myeloma progression remain largely undefined. MDM2 (murine double minute 2), an E3 ubiquitin ligase in the cell nucleus, is known for its role in regulating the tumor suppressor protein p53 [2]. Under normal cellular conditions, MDM2 keeps the level of p53 low by promoting its degradation via the ubiquitin-proteasome pathway [3,4,5]. This interplay between MDM2 and p53 is critical for the control of cell cycle arrest and apoptosis [6,7]. MDM2 is often overexpressed in various cancers, leading to the suppression of p53 and thus bypassing these protective mechanisms [2,8]. In addition to its interaction with p53 [9], MDM2 interacts with a variety of other cellular proteins, including transcription factors and signaling molecules [10]. For example, under sterile inflammatory conditions, MDM2 can act as a transcription factor for NF-κB, independent of p53 [11]. Some studies also suggest that MDM2 has pro-angiogenic effects that may contribute to tumor development [12,13]. Although limited data suggest that MDM2 is overexpressed in MM and is associated with disease progression and chemotherapy resistance [14], the role of MDM2 protein expression in MM has not been fully elucidated.

The NF-κB signaling pathway (Nuclear Factor Kappa-Light-Chain-Enhancer of Activated B Cells) is involved in the development and progression of MM, contributing to drug resistance [15]. The activation of the NF-κB signaling pathway is usually triggered by a variety of stimuli, including cytokines and various forms of cellular stress [16] that lead to its pro-inflammatory role associated with cancer development [17]. NF-κB is normally located in the cytoplasm of the cell, but upon activation, it migrates to the nucleus, where it regulates the expression of target genes that promote cell survival. This constitutive activation may also be due to genetic mutations and chromosomal translocations [18]. In addition, the dynamic interaction between myeloma cells and the bone marrow environment [19] influences NF-κB signaling through cytokine-, chemokine- and growth factor-mediated loops, promoting the proliferation, survival, migration, and drug resistance of myeloma cells [20].

Although the role of MDM2 and NF-κB has been extensively studied in malignancies, their role in MM remains poorly understood and there is virtually no data on a possible link between these proteins and MM.

The aim of this pilot study was to investigate the relationship between the immunohistochemical expression of MDM2 and NF-κB proteins in CD138-positive plasma cells before and after induction therapy with bortezomib-based therapies in patients with newly diagnosed MM, and to evaluate the potential association of these proteins with standard clinical parameters and selected prognostic factors.

## 2. Materials and Methods

This retrospective study included 42 newly diagnosed MM patients at the Clinical Hospital Centre Rijeka, identified according to the International Myeloma Working Group (IMWG) diagnostic criteria [21] from 2016 to 2023, and followed until the post-therapy period, i.e., the second trephine biopsy. Inclusion criteria for the study were: age ≥ 18 years, newly diagnosed MM patients treated with bortezomib-based first-line therapy, measurable disease, and available bone marrow trephine biopsy samples at the time of diagnosis and after induction therapy (8 cycles of treatment with bortezomib). Relapsed/refractory MM, newly diagnosed MM treated with other therapies or missing bone marrow trephine biopsy samples after treatment with bortezomib were not included in the study. We evaluated clinical and prognostic markers. Clinical markers- anemia (defined as haemoglobin value of >20 g/L below the lower limit of normal, or a haemoglobin value <100 g/L), hypercalcaemia (serum calcium >0.25 mmol/L higher than the upper limit of normal or >2.75 mmol/L) and renal insufficiency (defined as creatinine clearance <40 mL per min† or serum creatinine >177 μmol/L) were determined before and after therapy, as well as bone disease defined as one or more osteolytic lesions detected by X-ray or CT scan before therapy. Prognostic markers- beta-2-microglobulin (B2MG), albumin and lactate dehydrogenase (LD) were determined at the time of diagnosis and evaluated after the therapy. Notably, the International Staging System (ISS) stage that categorizes patients into three stages (ISS-1, ISS-2, and ISS-3) using two key markers: serum beta-2 microglobulin and serum albumin, was only evaluated at baseline, as is standard clinical practice at the time of diagnosis. Patients were treated with bortezomib-based induction therapy, VCD protocol (bortezomib, cyclophosphamide, dexamethasone) or VD protocol (bortezomib, dexamethasone) for eight cycles. Response to treatment was assessed using IMWG criteria [22] and categorized as complete remission (CR), very good partial remission (VGPR), partial remission (PR) or progressive disease (PD).

### 2.1. Immunohistochemistry

Bone marrow biopsies were obtained at the initial diagnosis and after the induction therapy. Tissue specimens were fixed in formalin and embedded in paraffin. Tissue slides were deparaffinized with xylene and then rehydrated in a graded series of ethanol solutions, followed by a transition to distilled water. Antigen retrieval was performed by heating the slides in a Tris/EDTA buffer (pH 9.0) in a microwave oven for 15 min at 100 °C. The primary antibodies were: Mouse monoclonal CD138 (clone MI15, M7228, DAKO Agilent, Glostrup, Denmark, PT Link, (dilution 1:100, incubation 30 min at room temperature); Mouse monoclonal MDM2 (clone IF2, 479M-96, Cell Marque, Rocklin, CA, USA, PT Link, dilution 1:50, incubation 30 min at room temperature); and rabbit monoclonal NF-κB, p65 (clone E379, ab32536, Abcam, Cambridge, UK, PT Link, dilution 1:500, incubation 30 min at room temperature). A small number of cases were stained as negative controls by replacing the primary antibody with a buffer solution (S3006, DAKO, Glostrup, Denmark). The DAKO EnVisionTM Doublestain System (K5361, DAKO, Glostrup, Denmark) was used to assess the expression of NF-κB and MDM2 in BM plasma cells using the dual immunohistochemical techniques CD138+/NF-κB and CD138+/MDM2. In this system, two antigens in the same sample are stained differently using an automated immunostainer with contrasting dyes: anti-CD138 is displayed as a red membrane stain for plasma cell identification (Chromogene Red, 960D, Sigma-Aldrich, St. Louis, MO, USA), while the expression of NF-κB or MDM2 was displayed brown as a nuclear positivity (DAB, ab64238, Abcam, Boston, MA, USA).

### 2.2. Evaluation of the Immunostaining

The slides were first scanned at low power to identify the areas with the greatest number of plasma cells. The percentage of positively stained plasma cells was assessed in relation to the total number of tumor plasma cells. The ratio before the treatment was calculated as the number of positive cells within a total of 100 plasma cells in at least three representative fields visible at 400× magnification. The number of plasma cells after the treatment was lower, so the ratio after the treatment was calculated as the number of positive cells within a total of 50 plasma cells in at least three representative fields visible at 400× magnification. The immunoreactivity of NF-κB and MDM2 was assessed by the percentage of plasma cells that were positive, as follows: negative expression, <10% positive plasma cells; low expression, 11–50% positive plasma cells; and high expression, >50% positive plasma cells. Each slide was scored independently, and all assessments were performed with the supervision of a hematopathologist/clinical cytologist (NJ and ISB) who evaluated the slides according to a double-blind protocol. In case of significant discrepancy, the average percentage of positive cells was calculated for statistical analysis. We did not use the histo-score in this study because the intensity of nuclear staining of MDM2 or NF-kB did not vary significantly.

### 2.3. Statistical Analysis

Statistical analyses were performed using the MedCalc software (v18.2.1). All tests were two-sided, and a significance level of *p* < 0.05 was used. Categorical variables are presented as whole numbers and proportions. The existence of a statistically significant difference between categorical variables was tested using the χ^2^ test. Quantitative variables are presented depending on the type of distribution the data follow. Age is expressed as the median, along with the minimum and maximum values. The normality of quantitative variables was tested using the Kolmogorov–Smirnov test, and the appropriate test was used to examine the difference between groups, depending on the normality of the distribution or the data presentation. For data that do not follow a normal distribution (i.e., data deviating significantly from the theoretical normal distribution), non-parametric tests were used, and the data are presented as the median and interquartile range. For data that follow a normal distribution, parametric tests were used and the data are presented as the mean ± standard deviation. Welch’s *t*-test was used to compare the means between groups with unequal variances. When examining the difference between the examined markers among the groups before and after the therapeutic protocol, depending on the distribution, a parametric Student’s *t*-test or a non-parametric Mann–Whitney test was used. The relationship between the data was determined using correlation analysis. The association of protein expression change with the change in prognostic markers, which showed a significant change in concentration or activity after therapy in the study, was examined by logistic regression. Regression analysis was performed stepwise. First, factors that were statistically significant in predicting the outcome were identified in the univariate regression analysis. For a variable to be statistically significant, the obtained *p* value must be <0.05. Each variable is also described using the OR (odds ratio) parameter with the associated 95% confidence interval. If OR > 1, then the tested variable increases the appearance of the tested outcome, and if OR < 1, the tested variable decreases the appearance of the outcome. If significant variables are identified in the univariate analysis, they are included in the multivariate regression model. Variables that remained significant in this model are considered statistically significant predictors of outcome.

### 2.4. Ethical Approval

Ethical approval was obtained from the Ethics Committee of the Clinical Hospital Center Rijeka, and all procedures were performed in accordance with the Declaration of Helsinki.

## 3. Results

The median age of the patients was 73.5 years (range 40–84) at the time of diagnosis, with slightly more women in the sample (*p* = 0.220). The majority of subjects were diagnosed with MM IgG kappa, 35% of patients had renal impairment, and osteolysis was present in 74% of cases. In addition, 76% of subjects received the VCD therapy protocol. 40% of patients achieved complete remission. More detailed characteristics of the cohort are presented in Table 1.

### 3.1. Results of Double Immunohistochemical Staining in the Bone Marrow

**Figure 1:**
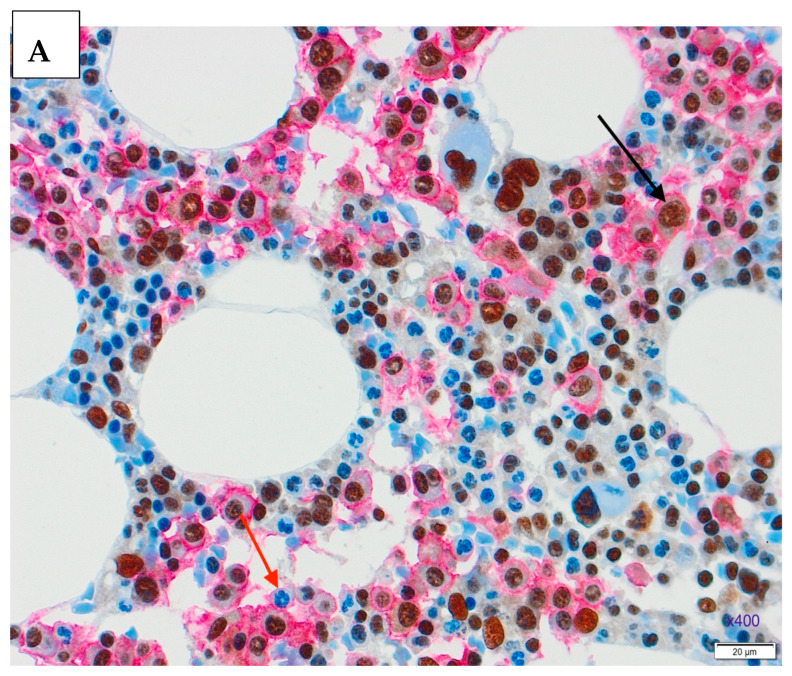

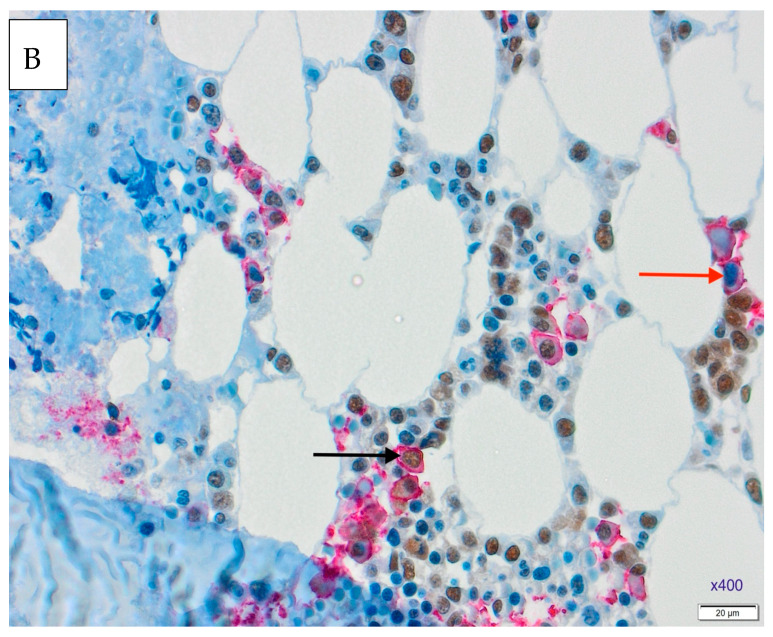
Double staining in the trephine biopsy, CD138 staining with Dako REAL Detection System Alkaline Phosphatase kit K5005, red chromogenic presentation, MDM2 staining with DAB chromogen in brown, ×400. (**A**). Newly diagnosed MM trephine biopsy, positive nuclear staining (black arrow) and negative nuclear staining (red arrow). (**B**). Trephine biopsy after the therapy, positive nuclear staining (black arrow) and negative nuclear staining (red arrow).

**Figure 2:**
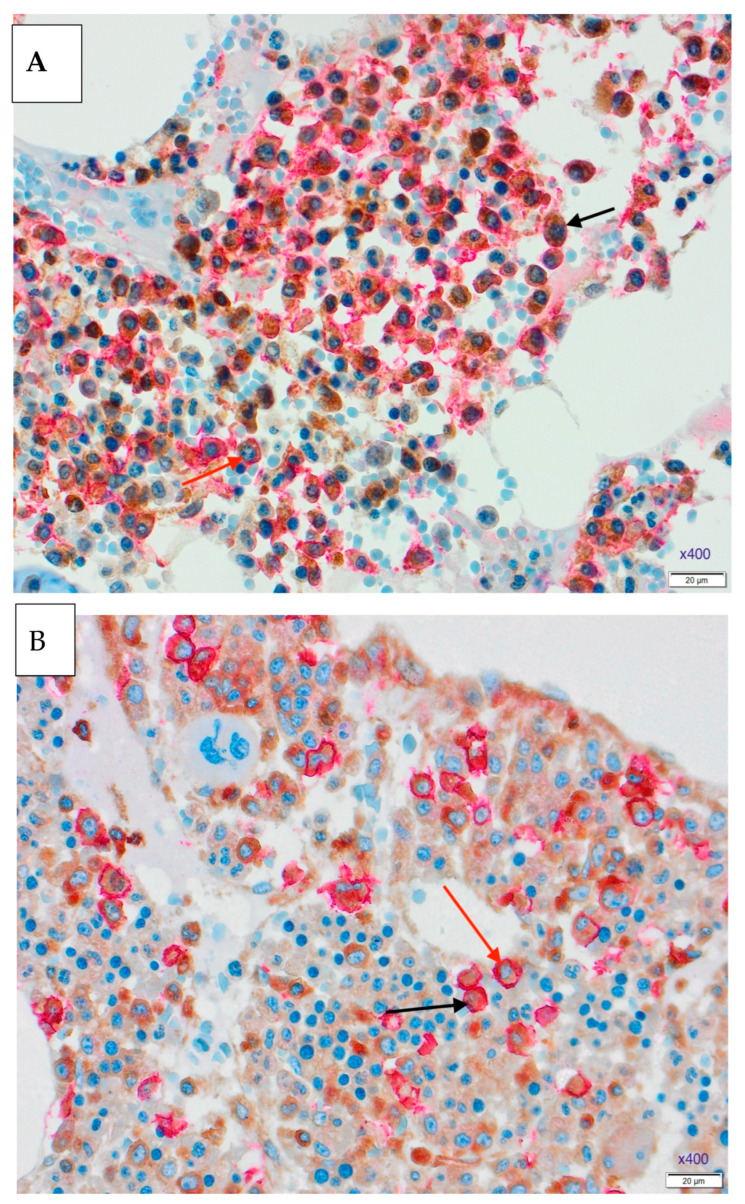
Double staining in the trephine biopsy, CD138 staining with Dako REAL Detection System Alkaline Phosphatase kit K5005, red chromogenic presentation, NFkB-p65 staining with DAB chromogen in brown, ×400. (**A**). Newly diagnosed MM trephine biopsy, positive nuclear staining (black arrow) and negative nuclear staining (red arrow). (**B**). Trephine biopsy after the therapy, positive nuclear staining (black arrow) and negative nuclear staining (red arrow).

Representative microscopic images of double staining of the markers in the trephine biopsy at the time of diagnosis and after the therapy are shown in Figure 1 and Figure 2.

### 3.2. Expressions of NF-κB and MDM2 Proteins Before and After Therapy

**Figure 3:**
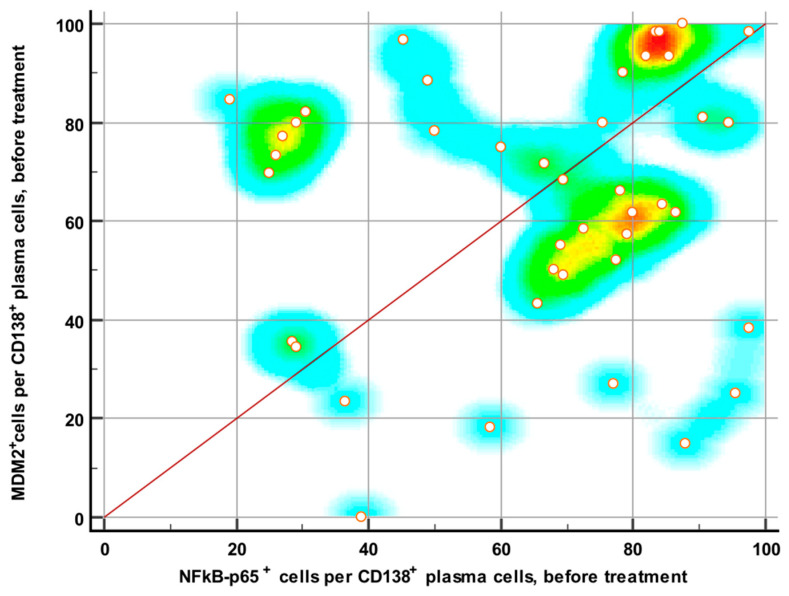
Scatter density plot of MDM2 vs. NF-κB protein expression in CD138^+^ plasma cells before therapy, with a regression line. The data are scattered and mostly point to the upper right corner, indicating high expression levels of MDM2 and NF-κB before treatment.

High expression (>50% positive plasma cells) and low expression (11–50% positive plasma cells) of NF-κB protein was found in 71.5% (30) and 28.5% (12) of biopsies at the time of diagnosis, respectively. There were no negative expressions (<11% positive plasma cells) of NF-κB protein at the time of diagnosis. High expression, low expression and negative expression of MDM2 protein was found in 73% (31), 23.8% (10) and 2.3% (1) of biopsies at the time of diagnosis, respectively. The statistically significant correlation between MDM2 and NF-κB expression before therapy was not observed (*p* = 0.412, R = 0.130 (−0.181–0.417)) (Figure 3).

**Figure 4:**
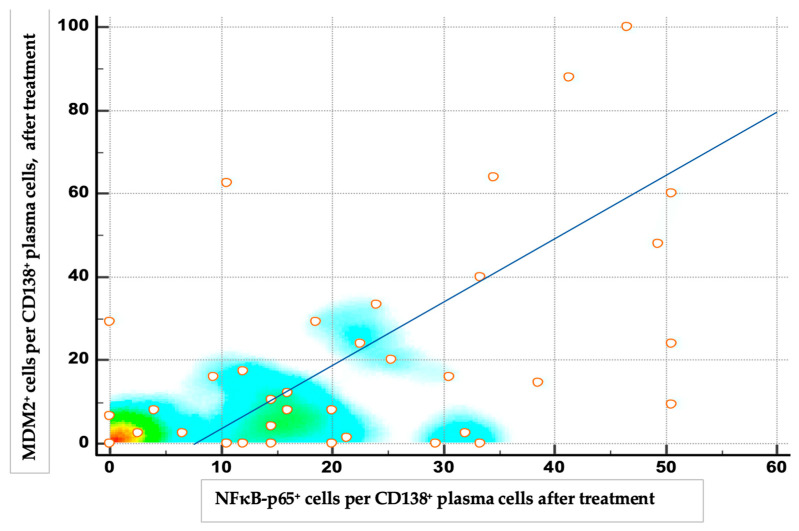
Scatter density plot of MDM2 vs. NF-κB protein expression in CD138^+^ plasma cells after therapy, with a regression line. Most data points cluster toward the lower left corner, indicating that most patients had low NF-κB and MDM2 expression levels post-treatment.

The expression of both proteins decreased significantly after the therapy (*p* < 0.001). High expressions, low expressions and negative expressions of NF-κB protein after the therapy were 4.8% (2), 66.6% (28) and 28.6% (12), respectively. High expressions, low expressions and negative expressions of MDM2 protein after the therapy were 11.9% (5), 33.3% (14) and 54,8% (23), respectively. The post-treatment percentages of MDM2-positive and NF-κB-positive plasma cells were positively correlated (r = 0.568, 95% confidence interval (CI) 0.320–0.744; *p* < 0.001), indicating that patients with higher residual NF-κB expression tended to also have higher MDM2 expression, and vice versa (Figure 4).

### 3.3. Expressions of NF-κB and MDM2 Proteins and Treatment Response

**Figure 5:**
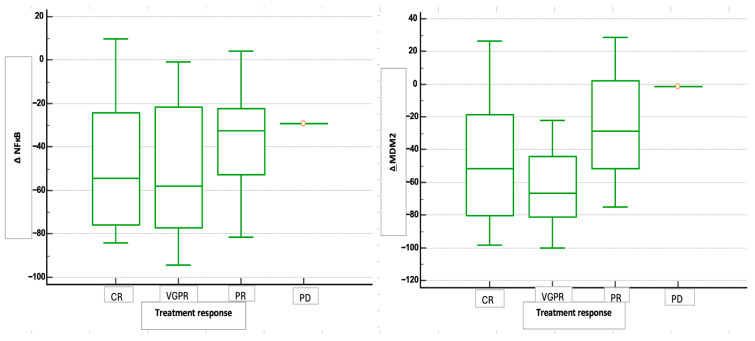
Changes in NFκB (ΔNFκB) and MDM2 expression (ΔMDM2) in relation to treatment response. The box plot shows the distribution of ΔNFκB values across four patient groups categorized by their response to therapy (CR—complete remission; VGPR—very good partial remission, PR—partial remission; PD—progressive disease). Groups CR and VGPR showed a more pronounced reduction in NFκB and MDM2 expression compared with groups PR and PD.

Although we found no statistically significant difference in the median value of NF-κB and MDM2 expression regarding response to therapy, results suggest that the decreases in NFκB and MDM2 expression were greatest in patients who responded best to therapy, i.e., who achieved complete remission and very good partial remission (Figure 5).

Furthermore, no significant differences were observed in ΔNFκB between response groups (*p* = 0.461), however ΔMDM2 showed a trend toward significance (*p* = 0.051) (Table 2).

For the one patient who exhibited progression, NF-κB and MDM2 expression levels were 80% and 61.6% before therapy, decreasing to 50.6% and 60.6% after therapy, respectively. This indicates that the patient with progressive disease had a smaller reduction in the expression of both proteins compared to patients who responded to therapy. The reduction in expression for both proteins is much lower than the median difference (Table 2), with the reduction in MDM2 expression for this patient who experienced PD being almost negligible.

### 3.4. NF-κB and MDM2 Proteins Expression and Clinical/Prognostic Parameters

The expression of both proteins significantly reduced after the therapy (*p* < 0.001). Although the clinical parameters improved after therapy, there was no statistically significant difference in median values of clinical parameters before and after therapy, except for a significant decrease in creatinine levels after therapy (*p* < 0.001). Regarding prognostic markers, there was a statistically significant increase in serum albumin and a decrease in B2MG. Although the median LD concentration was higher after therapy compared to pre-treatment levels, both median values remained within the reference range for this parameter (Table 3).

Univariate regression analysis showed that decreased NF-κB expression had a favorable outcome on creatinine concentration (OR (95% CI) 0.963 (0.927–0.999); *p* = 0.049). Multivariate regression analysis confirmed that decreased NF-κB expression is an independent prognostic factor for improved renal function (OR (95% CI) 0.979 (0.956–1.00); *p* = 0.017), which means that a decrease in NF-κB expression was associated with a favorable impact on creatinine levels. Our results showed no statistically significant correlation between changes in protein expression and other clinical parameters (haemoglobin, calcium), therefore they were not included in the univariate regression analysis. Furthermore, no significant association was found between the expression of the tested markers and the presence of osteolysis in the study participants at the time of diagnosis. Our results showed no statistically significant correlation between changes in protein expression and ISS stage (*p* > 0.05). Univariate regression analysis did not demonstrate that alterations in protein expression were significantly predictive of increases in albumin concentration or a reduction in B2MG or LD concentration (Table 4).

## 4. Discussion

Limited data suggest that MDM2 is overexpressed in MM and is associated with disease progression and chemotherapy resistance [14]. Faruq et al. suggested that MDM2 is associated with poor prognosis and drug resistance in human myeloma cell lines (HMCLs). It this study, inhibition of MDM2 increased the sensitivity of resistant HMCLs and primary MM xenograft samples to bortezomib and other anti-myeloma drugs, demonstrating that MDM2 can modulate drug response. However, the role of MDM2 protein expression in MM has not yet been well established. In contrast, the role of NF-κB is better understood. The most abundant form of NF-κB activated by pathologic stimuli is the p65: p50 heterodimer. Disproportionate increase in activated p65 is integral to the pathogenesis of many diseases. Thus, the NF-κB p65 signaling pathway has been a pivotal point for intense drug discovery and development [23]. Because of this, our goal was to investigate the possible role of this subunit in the pathogenesis of MM and determine its eventual association with MDM2 expression in tumor cells. The functional relationship between NF-κB and MDM2 may have significant implications for tumor cell survival. For example, in colorectal cancer, NF-κB has been suggested to be essential for activin-induced cancer cell migration via upregulation of the PI3K-MDM2 signaling pathway. This suggests that MDM2 expression can be increased by the recruitment of NF-κB p65 to the MDM2 promoter [24]. This possible positive feedback loop between NF-kB and MDM2, ultimately leading to tumor cell progression, should also be investigated in MM, as it offers therapeutic targeting opportunities, as described in the literature [25].

Using double immunohistochemical methods in trephine biopsies, we found that NF-κB and MDM2 proteins are highly expressed in newly diagnosed MM, with rates of 71% and 73%, respectively. The evidence of these two proteins in malignant plasma cells of MM patients at the protein level suggests their involvement in the pathogenesis of this neoplasm. The patients included in this study were diagnosed when bortezomib-based regimens were recommended as first-line therapy for MM. Similarly to other research studies [26,27] we found that bortezomib reduces NF-κB expression in MM; however, we found no previously published studies regarding MDM2 expression following bortezomib therapy. Bortezomib significantly reduces the expression levels of both proteins in our study group (*p* < 0.001). Our results suggest that the reduction in NFκB and MDM2 expressions were greatest in patients who experienced best response to therapy, e.i. CR and VGPR. Although we found no significant differences in changes in NFκB and MDM2 expressions between response groups (*p* = 0.461), the change in MDM2 expression showed a trend toward significance (*p* = 0.051). The one patient who experienced disease progression showed the smallest reduction in the expression of both proteins after the therapy, indicating possible association between reduction in NFkB and MDM2 expressions and response to therapy, but larger sample sizes are needed.

The correlation between these proteins in our study was not significant before treatment (*p* = 0.412), but we found a significant positive correlation between the expression of MDM2 and NF-κB proteins in CD138-positive plasma cells after induction therapy (*p* < 0.0001, r = 0.516), indicating that patients with higher residual NF-κB expression tended to also have higher MDM2 expression, and vice versa. Our results indicate, for the first time, a possible link between these two proteins in the pathogenesis of MM and treatment response.

We evaluated clinical and selected prognostic parameters with expression of these proteins before and after the therapy. Regarding prognostic markers, there was a statistically significant increase in serum albumin and a decrease in B2MG after the therapy, while median LD concentration remained within the reference range. Our results showed no statistically significant correlation between changes in protein expression and measured prognostic parameters (albumin, B2MG, LD) or ISS stage (*p* > 0.05).

Although the values of clinical parameters improved after therapy, there was no statistically significant difference before and after therapy, except for a significant decrease in creatinine levels after therapy (*p* < 0.002). Furthermore, no significant association was found between the expression of the tested proteins and the presence of osteolysis in the study participants at the time of diagnosis. While therapy positively affected clinical parameters, no statistically significant association between clinical parameters and tested proteins was found, except for favorable effect of NFkB reduction to renal function. Impaired renal function affects up to 50% of newly diagnosed MM patients. It is an independent predictor of poor survival with a higher risk of disease progression or death [28]. Restoration of renal function is one of the most important therapeutic goals in these patients [29]. Bortezomib-based regimens are the cornerstone of treatment for patients with MM and renal dysfunction at diagnosis [30]. Thirty-five percent of our patients experienced renal impairment and were treated with bortezomib. We found that bortezomib reduced NF-κB expression levels, which had a favorable effect on renal function in our study group. Therefore, NFκB-p65 has the potential to serve as a marker of renal function and therapeutic response. The rationale for the use of bortezomib (and possibly other proteasome inhibitors) in myeloma renal disease may arise from its potent inhibitory effect on NF-κB [31]. This transcription factor is strongly activated in the renal tubule cells of patients with proteinuria, and inhibition of NF-κB has been shown to reduce inflammation and fibrosis in an experimental model of glomerulonephritis [32]. This finding supports previous conclusions and improves our understanding of the mechanisms by which bortezomib affects renal function. In addition, other proteasome inhibitors such as ixazomib and carfilzomib, used in MM treatment, can also inhibit NF-κB signaling, highlighting the therapeutic importance of this pathway [17].

It has been suggested that MDM2 (among others) may be a useful prognostic indicator of bone lesions in primary bone tumors [33], but we have not established its association with osteolytic lesions in MM.

We recognize that this is a retrospective study with a small sample size and a selection bias towards subjects who responded to treatment and underwent post-treatment trephine biopsy. For most patients with progressive disease measurable by clinical parameters, we did not perform a biopsy but instead continued with second-line treatment, which is the reason for the very small number of respondents in the group of patients with progressive disease. Our study lacks other important prognostic factors, such as cytogenetics which unfortunately was not performed for technical reasons in our institution at the time of inclusion of the patients in this research. This pilot study was designed as an immunohistochemical study, and now that the first positive results are available, RT-PCR will be integrated in our further research in addition to a larger number of patients in order to make future results more robust.

The results of this study demonstrated for the first time the in vivo protein expression of MDM2 in the bone marrow of patients with MM, as well as the possible effect of bortezomib on the expression of this protein. The findings also indicate a mutual connection between these two pathogenetically important molecules in the bone marrow microenvironment of patients with MM after initial therapy, which warrants further investigation. Finally, a possible association between NF-κB and kidney damage was identified, which may be clinically significant, as kidney disease is a manifestation that largely determines the patient’s prognosis.

In future research, we intend to include larger sample sizes and new therapies that are advancing first-line treatment, such as daratumumab-based regimens. It would be interesting to evaluate expression levels and possible correlations between these proteins in relapsed and/or refractory MM patients or even extramedullary MM, with longer follow up periods to determine correlation with progression free survival and overall survival. Such studies that evaluate microenvironment of bone marrow in MM are necessary to fully clarify the underlying mechanisms and the potential therapeutic impact of targeting this signaling pathway in MM.

## 5. Conclusions

This pilot study demonstrated for the first time in vivo protein expression of MDM2 in the bone marrow of patients with multiple myeloma, as well as the possible effect of bortezomib on the expression of this protein in the microenvironment of multiple myeloma. We identified a high expression of MDM2 and NF-κB proteins in CD138-positive plasma cells in newly diagnosed MM in our study group. A significant reduction in the expression levels of both proteins was observed after bortezomib-based treatment. There is a positive correlation between the expression of MDM2 and NF-κB proteins after the therapy. Importantly, a decrease in NF-κB expression was associated with a favourable impact on creatinine levels, suggesting improved renal function in patients treated with bortezomib-containing regimens. These findings support the potential clinical relevance of MDM2 and NF-κB in MM and emphasise the need for further investigation in larger cohorts with longer follow-up to better understand their role in disease biology, response to treatment and long-term outcomes.

## Figures and Tables

**Table 1:** The characteristics of the cohort and response to treatment.

	Subjects N = 42 (%)
Gender	Male—17 (40.5) Female—25 (59.5)
Paraprotein type	IgG kappa—19 (45.3) IgG lambda—4 (9.5) IgA kappa—6 (14.3) IgA lambda—3 (7.1) FLC * kappa—3 (7.1) FLC * lambda—7 (16.7)
International staging system (ISS)	ISS I—14 (33.3) ISS II—10 (23.9) ISS III—18 (42.8)
Anemia	Present—21 (50.0) Absent—21 (50.0)
Hypercalcemia	Present—3 (7.2) Absent—39 (92.8)
Bone lesions	Present—31 (74.0) Absent—11 (26.0)
Renal impairment	Present—15 (35.7) Absent—27 (64.3)
Therapy	VCD *—32 (76.1) VD *—10 (23.8)
Response to treatment	CR *—17 (40.6) VGPR *—12 (28.5) PR *—12 (28.5) PD *—1 (2.3)

* FLC—free light chain; VCD—bortezomib, cyclophosphamide, dexamethasone; VD—bortezomib, dexamethasone; CR-complete remission; VGPR—very good partial remission; PR—partial remission, PD—progressive disease.

**Table 2:** Median percentage changes (Δ) in NFκB and MDM2 proteins expression according to treatment response.

Treatment Response	N = 42 (%)	Δ NFκB (%) Median (IQR)	Δ MDM2 (%) Median (IQR)
CR	17 (40.6)	−54.4 (−76.0–−24.4)	−51.7 (−80.5–−18.6)
VGPR	12 (28.5)	−58.1 (−77.4–−21.8)	−66.5 (−81–−44.44)
PR	12 (28.5)	−32.4 (−52.7–−22.5)	−28.6 (−51.8–2.0)
PD	1 (2.3)	−29.4 (29.4–−29.4)	−1.6 (−1.6–−1.6)
***p***	**/**	**0.461**	**0.051**

The table presents median percentage changes (Δ) and interquartile ranges (IQR) in NFκB and MDM2 expression levels across different treatment response groups: complete response (CR), very good partial response (VGPR), partial response (PR), and progressive disease (PD). Negative values indicate a reduction in protein expression compared with baseline levels. Statistical analysis was performed using non-parametric tests. No significant differences were observed in ΔNFκB between response groups (*p* = 0.461), whereas ΔMDM2 showed a trend toward significance (*p* = 0.051).

**Table 3:** Proteins expression, clinical and prognostic parameters of MM patients before and after therapy.

Parameter	Before Therapy Median (IQR)	After Therapy Median (IQR)	*p* *
NFkB (%)	71 (45.3–84)	16 (4–32)	**<0.001**
MDM2 (%)	68.95 (49–82)	8 (0–24)	**<0.001**
Haemoglobin (g/L) *	101.8 ± 22.7	118.2 ± 14.8	0.052
Creatinine (μmol/L)	91.5 (67–127)	75.0 (64–100)	**0.002**
Calcium (mmol/L)	2.35 (2.21–2.50)	2.30 (2.21–2.36)	0.079
LD * (U/L)	165 (137–194)	189.5 (176.0–206)	**0.039**
Albumin (g/L)	36.5 (33–42)	60 (49.1–63.6)	**<0.001**
B2MG * (mg/L)	4.3 (2.7–8.4)	3.0 (22–3.9)	**<0.001**

* *p*—Level of significance in difference before and after therapy (Wilcox signed-rank test for quantitative variables, Student’s *t*-test for hemoglobin, and McNemar’s test for qualitative variables)*;* B2MG—beta-2-microglobulin; LD—lactate dehydrogenase.

**Table 4:** Regression analysis results for protein expression changes and selected parameters.

Parameter	NFKB				MDM2	
	Univariate Regression		Multivariate Regression		Univariate Regression	
	OR (95%CI)	*p*	OR (95%CI)	*p*	OR (95%CI)	*p*
Creatinine (decrease)	0.963 (0.927–0.999)	0.049	0.979 (0.956–1.00)	0.017	1.016 (0.994–1.038)	0.159
Albumin (increase)	0.982 (0.942–1.024)	0.395	/	/	1.017 (0.991–1.045)	0.213
β2 microglobulin (reduction)	0.991 (0.961–1.022)	0.568	/	/	0.999 (0.978–1.020)	0.918
LD (reduction)	0.987 (0.958–1.017)	0.385	/	/	0.991 (0.970–1.011)	0.360

## Data Availability

Data supporting reported results can be found in Department of Hematology and Department of Pathology of the Clinical hospital center Rijeka, Croatia.
